# Effect of anti-SARS-CoV-2 BNT162b2 mRNA vaccination on thrombin generation in children with inflammatory bowel disease

**DOI:** 10.3389/fimmu.2023.1257072

**Published:** 2023-10-30

**Authors:** Vivien Stercel, Linda Lóczi, Orsolya Kadenczki, Éva Nemes, Béla Nagy, Rebeka Hodossy-Takács, Attila Ádám Szabó, Miklós Fagyas, János Kappelmayer, Tamás Szabó, Zsuzsa Bagoly

**Affiliations:** ^1^Department of Pediatrics, Faculty of Medicine, University of Debrecen, Debrecen, Hungary; ^2^Kálmán Laki Doctoral School, Faculty of Medicine, University of Debrecen, Debrecen, Hungary; ^3^Division of Clinical Laboratory Sciences, Department of Laboratory Medicine, Faculty of Medicine, University of Debrecen, Debrecen, Hungary; ^4^Hungarian Research Network (HUN-REN-UD) Cerebrovascular Research Group, Debrecen, Hungary; ^5^Division of Clinical Physiology, Department of Cardiology, Faculty of Medicine, University of Debrecen, Debrecen, Hungary

**Keywords:** COVID-19, Crohn’s disease, inflammatory bowel disease, ulcerative colitis, severe acute respiratory syndrome coronavirus-2, thrombin generation

## Abstract

**Background:**

Inflammatory bowel disease (IBD) including Crohn’s disease (CD) and ulcerative colitis (UC), are associated with higher thrombotic risk and enhanced thrombin generation (TG) in adults. Despite encouraging data reporting vaccine safety and low IBD flare rates in adults with IBD, vaccine hesitancy was demonstrated to be high in families of children with IBD. We aimed to find out whether TG is increased in children with IBD as compared to healthy controls and whether TG parameters show significant changes following SARS-CoV-2 mRNA vaccination.

**Patients and methods:**

In this observational case-control study, 38 children with IBD (CD:18, UC: 20) aged 12-18 years and 62 healthy age-and sex-matched children were enrolled. Blood was collected before the first dose and 2-6 weeks after the second dose of BNT162b2 (Pfizer-BioNTech) mRNA vaccine dose. Blood cell counts, fibrinogen, inflammatory markers (hsCRP, ferritin), anti-SARS-CoV-2 antibody levels were investigated, TG assay was carried-out using platelet-poor plasma. Detailed clinical parameters including disease activity scores (PUCAI, PCDAI) were registered pre-and post- vaccination. A guided questionnaire was used to collect data on adverse reactions (AEs) post- vaccination.

**Results:**

Baseline TG parameters did not differ between patients and controls. Endogenous thrombin potential showed a significant positive correlation with markers of inflammation and with PCDAI. Inflammatory parameters and TG did not increase in patients and controls post-vaccination. Vaccination significantly increased antibody levels in all three investigated groups, but post-vaccination anti-SARS-CoV-2 S IgG/IgM levels were below the 5^th^ percentile value of healthy children in more than one third of patients. Those receiving TNFα inhibitor therapy presented significantly lower SARS-CoV-2 S IgG/IgM levels as compared to patients on other immunosuppressive regimens. Systemic AEs did not differ between patients and controls while lower rate of local symptoms was found post-vaccination in children with IBD. Only 2 IBD flares were detected 2-6 weeks after the second dose of vaccination.

**Conclusion:**

Our study is the first to support the safety and efficacy of anti-SARS-CoV-2 BNT162b2 vaccination in children with IBD with detailed pre-and post-vaccination laboratory data including TG. Results of this study may further increase confidence and reduce vaccine hesitancy in caretakers of pediatric IBD patients.

## 1 Introduction

Inflammatory bowel disease (IBD) is an umbrella term used to define two chronic relapsing inflammatory intestinal disorders: Crohn’s disease (CD) and ulcerative colitis (UC) (1, 2). In both disorders, inflammation of the digestive system is found, but the affected areas of the gastrointestinal tract differ. Approximately 25% of patients with IBD are diagnosed before the age of 18 years (3). The clinical course of CD and UC includes episodes of flare with symptoms of diarrhea, abdominal pain, rectal bleeding, and weight loss, but extraintestinal symptoms may also occur. Patients with IBD are known to carry an increased risk for venous (VTE) and arterial thromboembolic events (ATE). The risk of VTE in patients with IBD has been reported to be related to disease activity and it is known to be further increased during active IBD flare and hospitalization (4). Although the exact pathomechanism of the thrombotic events in IBD patients have not been elucidated, it has been shown in a few studies, that the thrombin generation (TG) test, a global hemostasis assay to assess hyper- or hypocoagulability in plasma, might be of value to identify IBD patients at higher risk of thrombotic events (5, 6). The relationship between systemic inflammation, disease activity and the extent of thrombin generation (endogenous thrombin potential, ETP) has been confirmed in adults (5), and few studies were published in children with IBD (6, 7). In a cohort of children with CD, significantly higher ETP and higher peak thrombin values were reported during active disease as compared to healthy children (6). Moreover, a significant positive correlation between disease activity as assessed by the Pediatric Crohn’s Disease Activity Index (PCDAI) and thrombin generation parameters were described.

Following its discovery in 2019, the novel severe acute respiratory syndrome coronavirus-2 (SARS-CoV-2), responsible for the coronavirus disease 19 (COVID-19), has soon become a world-wide threat causing millions of deaths and substantial morbidity (8). Vaccination has been the most effective approach to reduce hospitalizations and deaths. As IBD patients were underrepresented in SARS-CoV-2 mRNA vaccine trials, and initial data on the efficacy and safety of vaccines in these patients were scarce, trust and willingness towards COVID-19 vaccination uptake was lower in IBD patients as compared to the general population (9). Despite encouraging data reporting vaccine safety and low IBD flare rates in adults with IBD, vaccine hesitancy was demonstrated to be high in families of children with IBD (10, 11). According to reports surveying caregivers of children with IBD, they were more concerned about vaccine safety and effectiveness than those with non-IBD diagnosis (10). Among other factors, this could be partially explained by a few reports indicating that adverse events post-vaccination, including IBD flare, were more common among younger patients and children (10, 12). Moreover, concerns about the risk of thrombotic events after receiving COVID-19 vaccines arose in the general population after the characterization of vaccine-induced immune thrombotic thrombocytopenia (VITT), a rare prothrombotic condition that has been associated with adenoviral vector-based COVID-19 vaccines (13). Although mRNA COVID-19 vaccines have not been linked to VITT, concerns about thrombosis after mRNA vaccination still persist.

In this study, we aimed to find out whether TG is increased in children with IBD as compared to healthy controls and whether TG parameters show significant changes following SARS-CoV-2 mRNA vaccination.

## 2 Materials and methods

### 2.1 Patients and controls

In this observational case-control study, children with IBD and healthy age-and sex-matched children receiving two doses of messenger ribonucleic acid (mRNA) vaccine BNT162b2 (Cominaty-Pfizer/BioNTech) were enrolled at the University of Debrecen, Department of Pediatrics. Vaccination of children 12 years or older by the mRNA vaccine BNT162b2 (Cominaty-Pfizer/BioNTech) was approved by the European Medicine Agency in May 2021, and in Hungary it commenced in June 2021. All participants received vaccination according to current protocols. Patient enrollment was initiated in June 2021 and was completed in May 2022. Inclusion criteria of patients were the following: age 12-18 years with previously diagnosed IBD (CD or UC), parental consent to receiving the Pfizer-BioNTech BNT162b2 vaccine and to participation in the study. Exclusion criteria included surgical intervention or thrombotic event in the past 3 months, history of hemorrhagic/thrombotic diathesis, other autoimmune diseases, malignant disease, anticoagulant therapy, anaemia or thrombocytopenia, hepatic- or renal disease. IBD was diagnosed by means of clinical, endoscopic, and histological findings in accordance with the consensus guidelines of ECCO/ESPGHAN (14). Inclusion criteria of healthy controls were: age 12-18 years, parental consent to receiving the Pfizer-BioNTech BNT162b2 vaccine and to participation in the study. Exclusion criteria: chronic disorders (malignancy, known haemorrhagic or thrombotic disorders, autoimmune disorders, chronic kidney or liver disease, diabetes mellitus, etc.) acute illness within the past 4 weeks, thrombotic event, surgery or major trauma within the past 3 months, medications including anticoagulant use. Baseline demographic data of participants (age, sex, BMI) and clinical characteristics of patients (disease activity, medications) were registered before the first vaccine dose. The clinical activity of CD was characterized by the Pediatric Crohn’s Disease Activity Index (PCDAI) (15). The Pediatric Ulcerative Colitis Activity Index (PUCAI) was used to characterize the clinical activity of children with UC (16). Follow-up of patients and controls took place at two to four weeks after the second vaccine dose. A guided questionnaire was used to collect data on adverse reactions (AEs) after each vaccination dose, conducted by a trained interviewer. Vaccine AEs were classified as localized or systemic reactions as described earlier (11). Localized AEs included local swelling, pain/sensitivity, redness or other local reactions at the injection site. Systemic AEs included fever, chills, fatigue, dizziness, headache, nausea, loss of appetite, joint pain, muscle pain, enlargement of lymph nodes, sleep disturbances, allergic reaction, rash, or other. If an AE was reported, the rate of severity was asked (mild: did not interfere with daily activity; moderate: required medication, interfered with daily activity; severe: hospitalization or medical care by professional was required). At follow up, all patients were assessed again for the clinical activity of IBD using PCDAI or PUCAI. Flare of IBD was defined as reported earlier (11), based on the increase of disease activity index, including the worsening of at least one of the following symptoms: abdominal pain, bowel movement frequency, rectal bleeding, extraintestinal manifestations.

### 2.2 Informed consent

The study design was in accordance with the guiding principles of the Declaration of Helsinki and was approved by the Institutional Ethics Committee of the University of Debrecen and the Ethics Committee of the National Medical Research Council. All parents or caregivers provided written informed consent.

### 2.3 Blood sampling and laboratory measurements

Venous blood was drawn on two occasions from the participants in this cohort: before the first dose of Pfizer-BioNTech BNT162b2 vaccination (up to 7 days before) and 2-6 weeks after the second vaccine dose (elapsed time 10.0±4.0 weeks). Routine laboratory tests (serum electrolytes, renal and liver function tests, lipid profile, serum iron, ferritin, high-sensitivity C-reactive protein measurement, complete blood count) were carried out immediately from the blood samples by standard laboratory methods (Roche Diagnostics, Mannheim, Germany and Sysmex Europe GmbH, Hamburg, Germany). For the examination of haemostasis tests, blood samples were collected to vacutainer tubes containing 0.109 M sodium citrate (Becton Dickinson, Franklin Lane, NJ) and were processed to obtain platelet-free plasma immediately (centrifugation twice at 1500 g, room temperature, 15 min). Screening tests of coagulation (prothrombin time, activated partial thromboplastin time, and thrombin time) were performed immediately on a BCS coagulometer using routine methods (Siemens Healthcare Diagnostic Products, Marburg, Germany). Fibrinogen levels were measured according to the method of Clauss on a BCS coagulometer (Siemens Healthcare Diagnostic Products, Marburg, Germany). Serum angiotensin convertase enzyme 2 (ACE2) activity was assessed using a specific quenched fluorescent substrate as described earlier (17). For the execution of thrombin generation assay, aliquots of citrated plasma were stored at −80°C until analysis.

### 2.4 Anti-SARS-CoV-2 antibody testing

For the quantitative determination of antibodies against SARS-CoV-2 nucleocapsid (N) and spike (S) proteins, two Elecsys^®^ anti-SARS-CoV-2 tests were used on an electro-chemiluminescent immunoassay instrument (Cobas^®^ 602, Roche Diagnostics, Mannheim, Germany). The anti-SARS-CoV-2 N assay uses a recombinant protein representing the nucleocapsid antigen in a double-antigen sandwich assay format. The test is intended as an aid in the determination of the specific humoral immune reaction to SARS-CoV-2. The anti-SARS-CoV-2 S assay uses a recombinant RBD protein in a double-antigen sandwich assay format, which favors the quantitative determination of high affinity antibodies against SARS-CoV-2. The test is intended as an aid to assess the adaptive humoral immune response, including neutralizing antibodies, to the SARS-CoV-2 S protein after natural infection with SARS-CoV-2 or in vaccine recipients. During the measurement, the total amount of anti- SARS-CoV-2 S immunoglobulins (IgG or IgM) was calibrated against the WHO international standard and expressed as binding antibody units (BAU)/mL. In case of anti-SARS-CoV-2 N assay, the result of a sample was given in the form of a cut-off index (COI; signal sample/cut-off). According to the manufacturer, seropositivity can be pronounced above the limit value of 0.8 BAU/mL of anti-SARS-CoV-2 S total Igs.

### 2.5 Thrombin generation measurements

Thrombin generation test was carried out from stored platelet-free plasma as described previously using the Thrombinoscope CAT (Calibrated Automated Thrombogram, Maastricht, The Netherlands) assay, based on the manufacturer’s instructions (Diagnostica Stago, Asnieres, France) (18). Briefly, 80 μL of plasma was incubated with 20 μL PPP-Reagent™ (containing 5 pM recombinant tissue factor and 4 μM phospholipids) in black round-bottomed 96-well microplates (Greiner Bio, One North America Inc., Monroe, MI, USA) for 10 minutes. For each sample, a calibrator (α2-macroglobulin-thrombin complex with known thrombin activity: Thrombin Calibrator™) was run in parallel, to correct the fluorescence signal for substrate consumption and to compensate plasma color variability. Thrombin generation was initiated by the addition of 20 μL of FluCa-Kit™ (fluorescent substrate and a buffer solution with CaCl_2_: Fluo Buffer). All measurements were performed in duplicates. Fluorescence was detected by a Fluoroskan Ascent^®^ fluorimeter (Thermo Fischer Scientific, Waltham, MA) and the TG curves were analysed by the Thrombinoscope software (Thrombinoscope BV, Maastricht, The Netherlands). Thrombin generation curves were characterised by the following parameters (calculated and presented by the Thrombinoscope software): 1/Lagtime: the moment from the initiation of the test until thrombin generation starts (the time needed for the first amounts of thrombin to be generated), 2/Endogenous Thrombin Potential (ETP): the area under the curve, 3/Peak Thrombin: the highest thrombin concentration formed, 4/Time to Peak: the time until the Peak Thrombin.

### 2.6 Statistical analysis

Statistical analysis was performed by Graphpad Prism 8.0 software (GraphPad Prism Inc., La Jolla, CA). Normality of data was studied using the Shapiro-Wilk and Kolmogorov-Smirnov tests. Normally distributed data is presented as mean ± standard deviation (SD), non-parametric data as median and interquartile range (IQR). For two-group analyses, Student’s t-test or Mann-Whitney U-test was performed, depending on the result of the normality tests. Wilcoxon signed-rank-test was used to analyse paired data sets. ANOVA using the Bonferroni correction or Kruskal-Wallis test using the Dunn’s post-hoc test were applied for multiple comparisons. Spearman’s or Pearson’s correlation coefficient was used to determine the strength of correlation between continuous variables. Differences between categorical variables were assessed by χ^2^ test or by Fisher’s exact where appropriate.

## 3 Results

### 3.1 Baseline characteristics and TG parameters in children with IBD and controls

One hundred children were included in the study, 38 IBD patients (18 CD and 20 UC) and 62 age- and sex-matched healthy controls. Baseline characteristics of the study cohort are reported in **Table 1**. Based on the anti-SARS-CoV-2 N and anti-SARS-CoV-2 S assay, seropositivity was found at similar rates in healthy controls (60%, n=37) as compared to children with IBD (48%, n=16; p=0.2813). Among the baseline routine laboratory parameters, CRP levels, platelet count, and fibrinogen levels were significantly higher in patients with IBD as compared to controls. ACE2 activity was also significantly elevated in children with IBD as compared to controls. Iron levels and haemoglobin concentrations were significantly lower in patients. Baseline TG parameters did not differ between patients and controls. Except for BMI, which was significantly lower in children with CD, baseline characteristics of CD and UC patients did not differ significantly (**Table 2**). Six children had active (mild or moderate) disease, while most children had clinically quiescent IBD as assessed by PCDAI (median: 5, IQR: 0-15) and PUCAI (median: 0, IQR: 0-8). Thrombin generation parameters did not differ between CD and UC patients at baseline. Seropositivity for anti-SARS-CoV-2 total Ig (IgG/IgM) was not associated with any baseline TG parameters in either group (data not shown). Most patients received 5-ASA as monotherapy or in combination with azathioprine and/or TNFα inhibitor therapy (**Table 2**).

**Table 1:** Baseline characteristics of the study population before first vaccine dose.

Variables	Patients	Controls	*p*
Number of individuals, *n*	38	62	–
Crohn’s disease/Ulcerative colitis, *n*	18/20	–	–
Age, y, mean ± SD	15.8 ± 1.9	16.5 ± 1.5	0.1117
Male sex, *n* (%)	23 (61)	26 (42)	0.0991
BMI, kg/m^2^, median (IQR)	20.4 (18.1-22.5)	20.9 (19.1-23.1)	0.2825
Anti-SARS-CoV-2 seropositivity, *n* (%)	16 (48)	37 (60)	0.2813
Anti-SARS-CoV-2 N total Ig (IgG/IgM), COI	0.1 (0.1-9.1)	8.6 (0.1-36.5)	0.0098
Anti-SARS-CoV-2 S total Ig (IgG/IgM), BAU/mL	12.4 (0.4-158.3)	73.0 (0.4-206.3)	0.4814
Laboratory parameters, median (IQR) or mean ± SD			
hsCRP, mg/L	1.67 (0.58-11.92)	0.49 (0.49-0.76)	<0.0001
Iron, µmol/L	10.3 (5.6-16.4)	16.4 (12.7-21.1)	0.0003
Ferritin, µg/L	35.0 (17.1-54.8)	41.9 (24.7-67.6)	0.1540
WBC, G/L	6.7 (5.4-8.0)	6.4 (5.3-7.7)	0.6469
RBC, T/L	4.7 (4.5-4.9)	4.7 (4.5-5.0)	0.5326
Hemoglobin, g/L	132 ± 19	141 ± 12	0.0101
Platelet count, G/L	302 (247-370)	247 (217-291)	0.0005
PT, s	8.8 ± 0.5	9.0 ± 0.6	0.2025
APTT, s	29.4 ± 2.5	29.0 ± 2.6	0.5143
Fibrinogen, g/L	3.4 (2.8-3.9)	2.8 (2.4-3.4)	0.0002
ACE2 activity, mU/L	15.9 (14.5-19.4)	14.7 (12.8-16.8)	0.0142
Thrombin generation parameters, median (IQR) or mean ± SD			
Lag time (min)	3.0 (2.7-3.2)	2.9 (2.5-3.1)	0.0820
ETP (nM*min)	1720 (1399-2245)	1564 (1545-1985)	0.4243
Peak thrombin (nM)	289.1 (274.2-406.8)	322.8 (291.0-395.2)	0.4954
Time to peak (min)	5.7 ± 0.6	5.5 ± 0.6	0.2123

Continuous variables are expressed as mean ± SD or median (interquartile range). Categorical variables are indicated as number (percentage). ACE, angiotensin-converting enzyme; anti-SARS-CoV-2 N, anti-SARS-CoV-2 nucleocapsid antibody; anti-SARS-CoV-2 S, anti-SARS-CoV-2 spike protein antibody, APTT, activated partial thromboplastin time; BMI, body mass index; COI, cut-off index; ETP, endogenous thrombin potential; hsCRP, high sensitivity C-reactive protein measurement; IQR, interquartile range; n, number; PT, prothrombin time; RBC, red blood cell; SARS-CoV-2, severe acute respiratory syndrome coronavirus-2; SD, standard deviation; WBC, white blood cell; y, year. Anti-SARS-CoV-2 S antibody levels were not available in 4 patients (1 CD, 3 UC) and 1 healthy control.

**Table 2:** Baseline characteristics of patients before first vaccine dose.

Variables	Crohn’s disease	Ulcerative colitis	*p*
Number of individuals, *n*	18	20	–
Age, y, mean ± SD	15.8 ± 1.8	15.9 ± 2.1	0.9762
Male sex, *n* (%)	12 (66)	11 (55)	0.5216
BMI, kg/m^2^, mean ± SD	19.3 ± 3.1	22.3 ± 4.2	0.0185
Active disease, *n* (%)	2 (11)	4 (20)	0.6630
Disease activity, median (IQR)			
PCDAI	5 (0-15)	–	–
PUCAI	–	0 (0-8)	–
Anti-SARS-CoV-2 seropositivity, *n* (%)	11 (64)	5 (30)	0.0844
Anti-SARS-CoV-2 N total Ig (IgG/IgM), COI	0.2 (0.1-10.8)	0.1 (0.1-11.3)	0.4219
Anti-SARS-CoV-2 S total Ig (IgG/IgM), BAU/mL	3.4 (0.4-35.4)	0.3 (0.3-43.7)	0.3681
Laboratory parameters at baseline, median (IQR) or mean ± SD			
hsCRP, mg/L	3.3 (1.1-23.9)	1.1 (0.5-3.3)	0.0678
Iron, µmol/L	10.3 (4.9-15.6)	11.4 (5.7-17.23)	0.4632
Ferritin, µg/L	40.6 (17.1-91.1)	29.4 (16.6-44.6)	0.2346
WBC, G/L	6.8 (5.7-8.1)	5.8 (4.7-7.9)	0.2481
RBC, T/L	4.7 (4.5-5.2)	4.6 (4.5-4.8)	0.6087
Hemoglobin, g/L	137 (108-145)	132 (129-143)	0.6928
Platelet count, G/L	282 (247-356)	315 (246-384)	0.4828
PT, s	8.8 ± 0.5	8.8 ± 0.5	0.9924
INR	1.03 ± 0.05	1.02 ± 0.05	0.9134
APTT, s	30.2 ± 3.0	28.8 ± 1.8	0.0945
Fibrinogen, g/L	3.5 (2.8-5.3)	3.4 (2.8-3.9)	0.5604
ACE2, mU/L	16.2 ± 3.3	16.8 ± 3.6	0.5673
Thrombin generation parameters, median (IQR) or mean ± SD			
Lag time (min)	3.1 ± 0.3	2.9 ± 0.3	0.2215
ETP (nM*min)	1813 ± 550	1860 ± 475	0.7796
Peak thrombin (nM)	282.7 (270.2-414.0)	365.0 (283.8 – 410.8)	0.2304
Time to peak (min)	5.7 (5.0-6.0)	5.7 (5.4-6.1)	0.7802
Therapy, *n* (%)			
5-ASA	4 (22)	5 (25)	>0.9999
Azathioprine	1 (5)	1 (5)	>0.9999
5-ASA + Azathioprine	4 (22)	10 (50)	0.1008
5-ASA + TNFα inhibitor	2 (11)	0 (0)	0.2176
5-ASA + Azathioprine+ TNFα inhibitor	3 (16)	2 (10)	0.6525
5-ASA + Azathioprine + TNFα inhibitor + Glucocorticoid	1 (5)	1 (5)	>0.9999
5-ASA + Azathioprine + Glucocorticoid	1 (5)	0 (0)	0.4737
Azathioprine + Glucocorticoid	1 (5)	0 (0)	0.4737
Azathioprine + TNFα inhibitor	1 (5)	1(5)	>0.9999

Continuous variables are expressed as mean ± SD or median (interquartile range). Categorical variables are indicated as number (percentage). ACE, angiotensin-converting enzyme; ASA, acetylsalicylic acid; APTT, activated partial thromboplastin time; BMI, body mass index; COI, cut-off index; ETP, endogenous thrombin potential; hsCRP, high sensitivity C-reactive protein measurement; INR, international normalized ratio; IQR, interquartile range; n, number; PT, prothrombin time; PCDAI, Pediatric Crohn's Disease Activity Index; PUCAI, Pediatric Ulcerative Colitis Activity Index; RBC, red blood cell; SARS-CoV-2, severe acute respiratory syndrome coronavirus 2; SD, standard deviation; WBC, white blood cell; y, year. Anti-SARS-CoV-2 S antibody levels were not available in 1 CD and 3 UC patients.

Although the extent of TG was not significantly different between patients and healthy controls, in patients with IBD, ETP showed a significant positive correlation with markers of inflammation including hsCRP, WBC count, platelet count and fibrinogen and a significant negative correlation with hemoglobin levels at baseline (**Table 3**). Such associations were not found in healthy control children (**Supplementary Table 1**). In both groups, the extent of TG (ETP and peak thrombin) negatively correlated with PT and APTT (**Table 3**, **Supplementary Table 1**).

**Table 3:** Correlations between baseline anthropometric data, laboratory parameters and thrombin generation parameters in patients at baseline (before Pfizer-BioNTech BNT162b2 vaccine).

		Age (y)	BMI (kg/m^2^)	hsCRP (mg/L)	Ferritin (µg/L)	WBC (G/L)	RBC (T/L)	HGB (g/L)	PLT (G/L)	PT (s)	APTT (s)	Fibrinogen (g/L)
Thrombin generation parameters	Lag time (min)	r= -0.0457 95% CI: -0.3687 to 0.2870 p=0.7849	r= -0.0102 95% CI: -0.3376 to 0.3193 p=0.9513	r= 0.3677 95% CI: 0.0393 to 0.6242 p=**0.0252**	r= 0.1332 95% CI: -0.2089 to 0.4463 p=0.4319	r= 0.0905 95% CI: -0.2452 to 0.4069 p=0.5889	r= 0.0823 95% CI: -0.2529 to 0.4000 p=0.6231	r= -0.0247 95% CI: -0.3503 to 0.3062 p=0.8830	r= 0.1362 95% CI: -0.2013 to 0.4447 p=0.4149	r= -0.2764 95% CI: -0.5652 to 0.0728 p=0.1080	r= -0.0615 95% CI: -0.3910 to 0.2818 p=0.7212	r= 0.2894 95% CI: -0.0532 to 0.5711 p=0.0869
	ETP (nM*min)	r= 0.0607 95% CI: -0.2641 to 0.3732 p=0.7172	r= 0.2385 95% CI: -0.0878 to 0.5187 p=0.1493	r= 0.3274 95% CI: 0.0038 to 0.5890 p=**0.0479**	r= 0.3066 95% CI: -0.0193 to 0.5736 p=0.0649	r= 0.3841 95% CI: 0.0734 to 0.6268 p=**0.0173**	r= -0.1112 95% CI: -0.4161 to 0.2162 p=0.0123	r= -0.3433 95% CI: -0.5974 to -0.0265 p=**0.0348**	r= 0.5471 95% CI: 0.2756 to 0.7377 p=**0.0004**	r= -0.3410 95% CI: -0.6054 to -0.0087 p=**0.0450**	r= -0.4275 95% CI: -0.6629 to -0.1151 p=**0.0093**	r= 0.3503 95% CI: 0.0245 to 0.6088 p=**0.0362**
	Peak thrombin (nM)	r= 0.1125 95% CI: -0.2242 to 0.4252 p=0.5013	r= 0.0415 95% CI: -0.2909 to 0.3650 p=0.8046	r= 0.1766 95% CI: -0.1661 to 0.4812 p=0.2958	r= -0.2160 95% CI: -0.5121 to -0.1259 p=0.1991	r= 0.2640 95% CI: -0.0705 to 0.5452 p=0.1092	r= -0.1248 95% CI: -0.4354 to 0.2124 p=0.4555	r= -0.2752 95% CI: -0.5536 to 0.0585 p=0.0945	r= 0.4449 95% CI: 0.1363 to 0.6747 p=**0.0051**	r= -0.1936 95% CI: -0.5026 to 0.1592 p=0.2650	r= -0.5101 95% CI: -0.7231 to -0.2085 p=**0.0015**	r= 0.3547 95% CI: 0.0195 to 0.6182 p=**0.0338**
	Time to peak (min)	r= 0.0340 95% CI: -0.2977 to 0.3585 p=0.8393	r= 0.0283 95% CI: -0.3030 to 0.3535 p=0.8660	r= 0.0468 95% CI: -0.2905 to 0.3739 p=0.7829	r= 0.0891 95% CI: -0.2512 to 0.4098 p=0.6000	r= -0.1331 95% CI: -0.4422 to 0.2043 p=0.4257	r= 0.1937 95% CI: -0.1440 to 0.4909 p=0.2440	r= 0.2238 95% CI: -0.1130 to 0.5144 p=0.1769	r= 0.1183 95% CI: -0.4300 to 0.2187 p=0.4795	r= -0.2518 95% CI: -0.5470 to 0.0990 p=0.1445	r= 0.1270 95% CI: -0.2200 to 0.4454 p=0.4605	r= -0.0897 95% CI: -0.4147 to 0.2555 p=0.6027

Spearman or Pearson correlation. Bold values represent statistically significant associations. APTT, activated partial thromboplastin time; BMI, body mass index; CI, confidence interval; ETP, endogenous thrombin potential; HGB, hemoglobin; hsCRP, high sensitivity C-reactive protein measurement; PT, prothrombin time; RBC, red blood cell count; PLT, platelet count; WBC, white blood cell count.

### 3.2 The relationship between BNT162b2 mRNA vaccination and routine laboratory parameters, TG and IBD activity in the cohort 

**Figure 1:**
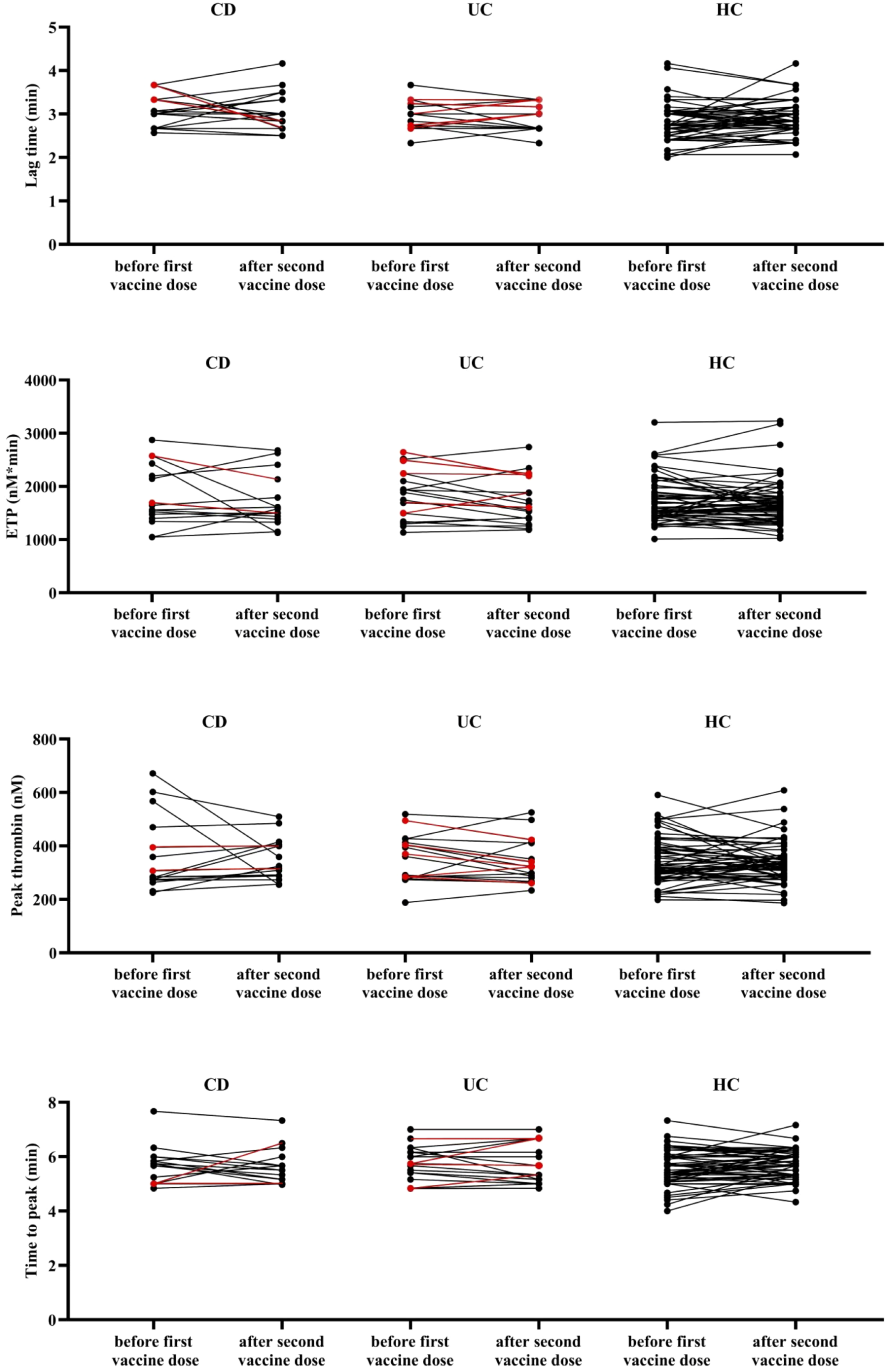
Thrombin generation parameters of the study cohort. Lag time, endogenous thrombin potential, peak thrombin, and time to peak parameters are shown in patients with CD, UC and healthy control children before (up to 7 days) the first dose of BNT162b2 mRNA vaccination *vs*. after (2-6 weeks) the second dose of vaccination. Red symbols represent patients with active disease at the given time point. CD, Crohn’s disease; ETP, endogenous thrombin potential; HC, healthy control; UC, ulcerative colitis. Paired Student’s t test or Wilcoxon signed-rank test was applied based on normality and no difference was found between paired data.

Routine laboratory parameters did not differ significantly between the samples obtained up to 7 days before the first dose of BNT162b2 mRNA vaccination and 2-6 weeks after the second dose in either group, except for a marginal decrease in fibrinogen and ACE2 levels in controls (**Supplementary Tables 2**–**4**). TG parameters of the cohort before and after vaccination are shown on **Figure 1**. Although in some cases individual changes in TG parameters could be observed over time, paired data were not significantly different in any group.

**Figure 2:**
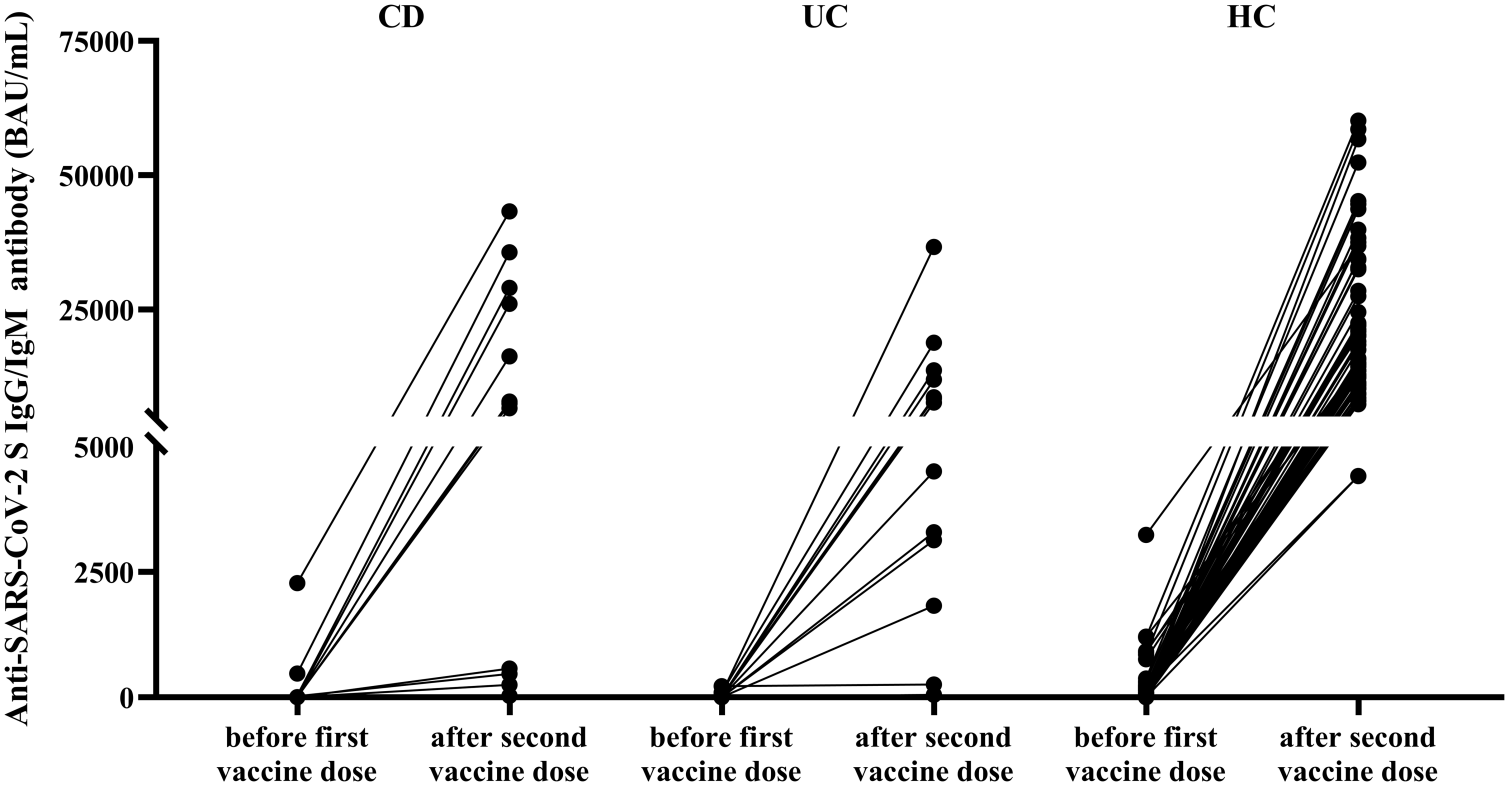
Anti-SARS-CoV-2 S IgG/IgM antibody levels in the studied cohort pre- and post-vaccination. Anti-SARS-CoV-2 S (spike protein) antibody levels are shown in patients with CD, UC and healthy control children before (up to 7 days) the first dose of BNT162b2 mRNA vaccination *vs*. after (2-6 weeks) the second dose of vaccination. CD, Crohn’s disease; HC, healthy control; UC, ulcerative colitis. Wilcoxon signed-rank test showed significant differences between paired data in all groups (p=0.0005, p=0.0002 and p<0.0001 for CD, UC and healthy controls, respectively). Anti-SARS-CoV-2 S antibody levels were not available in 1 CD, 3 UC patients and 1 healthy control.

**Figure 3:**
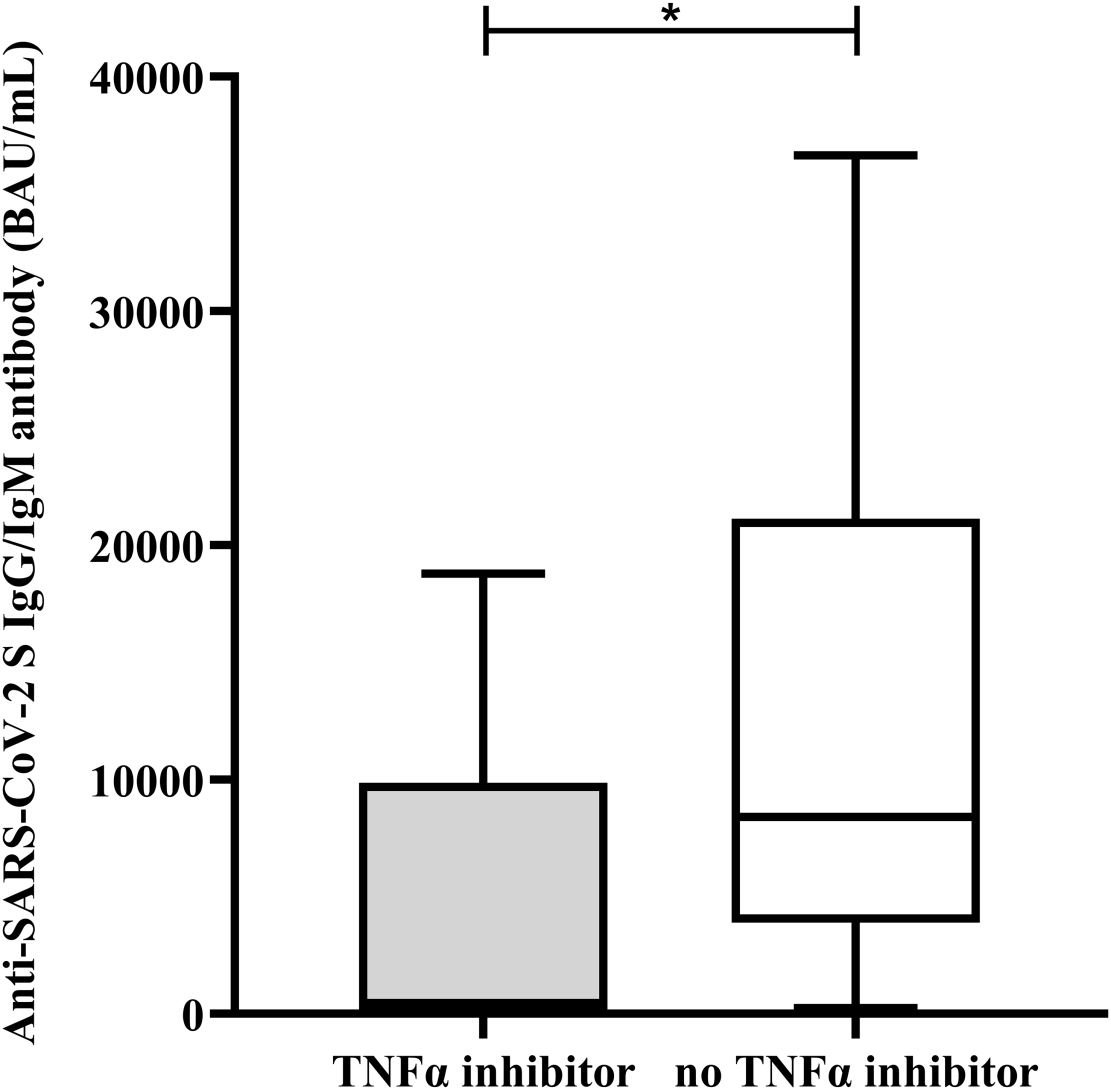
Anti-SARS-CoV-2 S IgG/IgM antibody levels 2-6 weeks after the second dose of BNT162b2 mRNA vaccination in IBD patients receiving or not receiving TNFα inhibitors. Anti-SARS-CoV-2 S (spike protein) antibody levels 2-6 weeks after the second dose of BNT162b2 mRNA vaccination are shown in IBD patients receiving (grey column) or not receiving (empty column) TNFα inhibitor therapy. The lower and upper box boundaries represent the 25^th^ and 75^th^ percentiles, respectively, horizontal solid lines represent the median, and whiskers indicate range. *p < 0.05.

According to the anti-SARS-CoV-2 S total Ig (IgG/IgM) measurement, vaccination significantly increased antibody levels in all three investigated groups (**Figure 2**, **Supplementary Tables 2**–**4**). In healthy control children, the obtained response was significantly higher as compared to children with CD and UC (anti-SARS-CoV-2 S total Ig median:15057; IQR: 11344-30449 *vs*. median:7475; IQR: 358-27495 and median:7606; IQR: 2480-12781 BAU/mL, respectively, p=0.0005). No significant difference was observed in post-vaccination anti-SARS-CoV-2 S IgG/IgM levels between CD and UC patients (p=0.9999). Postvaccination anti-SARS-CoV-2 S IgG/IgM levels were below the 5^th^ percentile value of healthy children in more than one third of patients (CD: 37.5%, n=6/16 and UC: 41.1%, n=7/17). Patients receiving TNFα inhibitor therapy presented significantly lower SARS-CoV-2 S IgG/IgM levels as compared to those receiving other immunosuppressive regimens (**Figure 3**).

**Figure 4:**
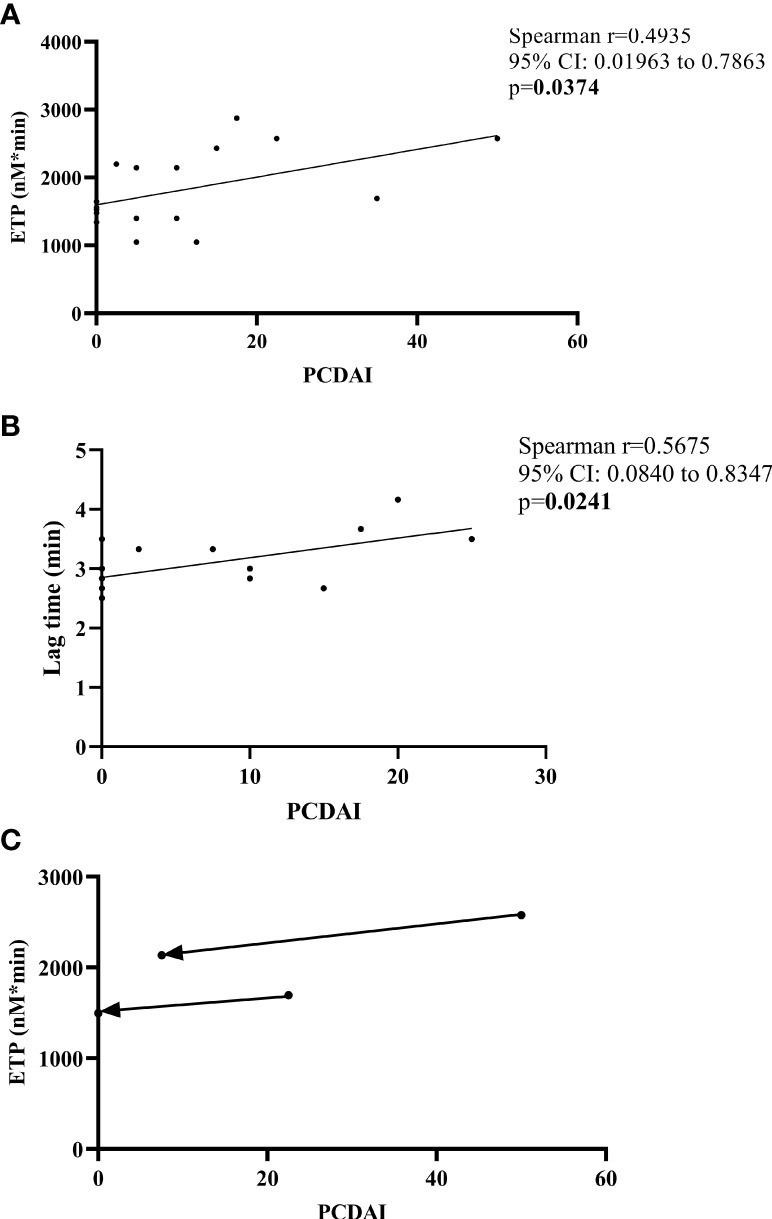
Correlation between thrombin generation parameters and disease activity (PCDAI) in CD patients. Correlations between ETP and PCDAI before first dose of BNT162b2 mRNA vaccination **(A)**, lag time and PCDAI 2-6 weeks after the second dose of vaccination **(B)** are shown. Changes in ETP *vs*. PCDAI are demonstrated in two CD patients with a decrease in activity post-vaccination **(C)**. Arrows point from pre-vaccination towards post-vaccination result. CI, confidence interval; ETP, endogenous thrombin potential; PCDAI, Pediatric Crohn’s Disease Activity Index.

IBD disease activity scores did not increase significantly following vaccination (**Supplementary Tables 3** and **4**). In children with CD, ETP showed significant association with PCDAI before vaccination (**Figure 4A**). After vaccination, only the lag time parameter of TG showed significant association with PCDAI (**Figure 4B**), although this correlation was not present before vaccination. None of the CD patients experienced disease flare within 6 weeks of vaccination, on the contrary, in two patients, a decrease in disease activity was observed post-vaccination, that was accompanied by a decrease in ETP (2575 to 2134 nM*min and 1693 to 1497 nM*min) (**Figure 4C**, **Supplementary Table 3**). In these two cases, glucocorticoid therapy was initiated in addition to the previous immunosuppressive treatment before the first blood draw, and the decrease in disease activity is most probably attributed to the intensified treatment. Two UC patients had IBD flare (one already with active IBD before vaccination) while one UC patient had a decrease in disease activity post-vaccination, but these changes were not accompanied by parallel changes in TG parameters (data not shown).

**Figure 5:**
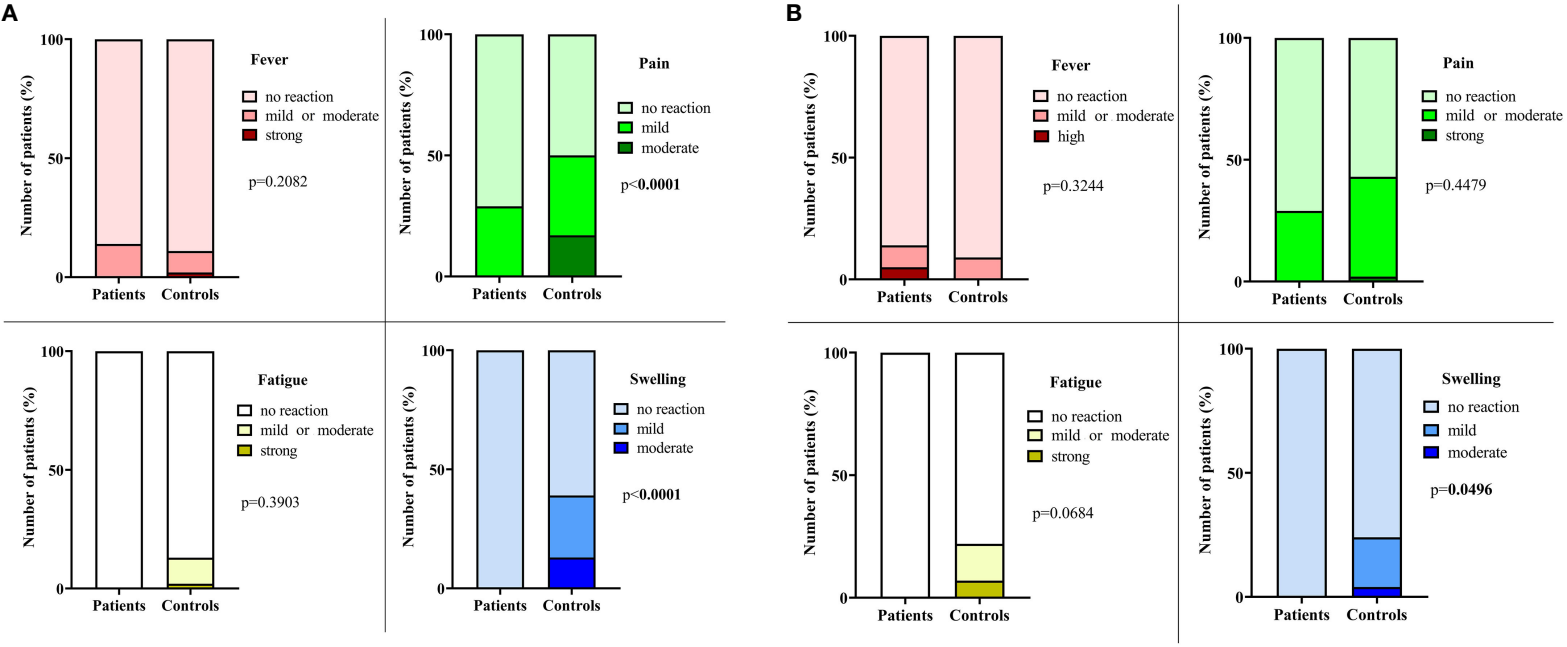
Systemic and local AEs in children with inflammatory bowel disease and healthy age- and sex-matched controls after BNT162b2 mRNA vaccination. Most common systemic (fever, fatigue) and local (pain, swelling) postvaccinal adverse reactions, self-reported by a questionnaire after the first dose **(A)** and the second dose **(B)** of BNT162b2 mRNA vaccination in IBD patients and healthy controls.

Systemic AEs, after the first dose and the second dose of BNT162b2 mRNA vaccination as self-reported by a questionnaire, did not differ between patients and controls (**Figures 5A, B**). Interestingly, local symptoms (pain, swelling) were found to be significantly less frequent in children with IBD as compared to healthy control children after the first and second dose of the vaccine (**Figures 5A, B**). No thrombotic events occurred in the cohort during the study period.

## 4 Discussion

To the best of our knowledge, this is the first study to investigate thrombin generation and other routine laboratory parameters including hemostasis screening tests, fibrinogen and inflammatory markers in pediatric patients with IBD before and after vaccination against SARS-CoV-2. Except for a marginal decrease in fibrinogen and ACE2 levels in controls, routine laboratory parameters and thrombin generation did not show significant alterations when comparing blood samples obtained before and after vaccination in IBD patients and in controls. Systemic AEs did not differ between patients and controls while lower rate of local symptoms was found post-vaccination in children with IBD. This is in line with previous studies in which decreased rate of post-vaccination AEs have been reported in pediatric patients with immune-mediated inflammatory disease taking immunosuppressive medication as compared to healthy subjects (19). In this cohort, only 2 IBD flares were detected 2-6 weeks after the second dose of BNT162b2 mRNA vaccination, while in 3 cases, disease activity was lower post-vaccination. These results support previous data (19–22) and provide reassurance that BNT162b2 mRNA vaccination in paediatric IBD patients is safe and well-tolerated. Our report is the first to support the safety of mRNA vaccination in a paediatric cohort with detailed laboratory data including ACE2 activity, inflammatory parameters and thrombin generation pre- and post-vaccination. Such studies demonstrating the safety of available vaccines are important as negative perceptions including false beliefs regarding the risk of thrombosis related to mRNA vaccination can be a major contributor to vaccine hesitancy.

Although the extent of TG did not differ between IBD and healthy children in this cohort, ETP showed a significant positive correlation with markers of inflammation including hsCRP, WBC count, platelet count, fibrinogen and a significant negative correlation with hemoglobin levels at baseline. The reason why the extent of TG was not significantly different in patients and controls might be related to the low rate of patients with active disease in this cohort. Notably, in patients with CD, ETP was not only associated with markers of inflammation but with disease activity (PCDAI) as well. This association disappeared at the second blood draw when all CD patients were in remission and only the lag time parameter showed association with PCDAI. Such association between the lag time parameter and PCDAI has been shown in a previous study in pediatric CD patients (6), which may indicate a delayed onset of thrombus formation and its potential implications merit further analysis. In two CD patients with a decrease in disease activity, ETP showed a parallel decrease. These results are also in line with previous observations demonstrating an association between CD activity and the extent of hypercoagulability (6). Only two previous reports investigated TG in pediatric IBD patients, with contradictory results published on the extent of TG in quiescent disease state (6, 7). Our results support the findings of Bernhard et al. (6), implying that ETP is not higher in inactive disease as compared to controls. Similarly to our results, Bernhard et al. found a positive association between ETP and PCDAI, and they report that lowering disease activity index decreases ETP (6). Identical findings have been reported in adult CD patients (5). Such results underline the cross-talk between inflammation and coagulation and indirectly support observations of the relationship between hypercoagulation and active disease. Interestingly, in this pediatric cohort of UC patients, although ETP was associated with laboratory markers of inflammation, it was not associated with disease activity score (PUCAI), which might be due to the overall high rate of patients with quiescent disease and a median PUCAI score of 0 in our cohort.

Previous SARS-CoV-2 infection, as demonstrated by seropositivity against SARS-CoV-2 S or N proteins, did not have an effect on TG in any of the investigated groups, which argues against a post-COVID hypercoagulable state in children with IBD and in healthy control children as well.

As demonstrated by others, the BNT162b2 mRNA vaccine was effective in this paediatric IBD patient group and seroconversion was as high as 100% (20, 21, 23). However, the anti-SARS-CoV-2 S antibody levels were significantly lower as compared to that observed in age- and sex-matched healthy control children. In agreement with previous findings, humoral response to vaccination was significantly influenced by anti-TNFα treatment in this cohort (21–23). Therefore, although our results confirm the efficacy of vaccination in pediatric IBD patients, at the same time our data highlight the need to remain vigilant about COVID-19 infections for those on combined immunosuppressive regiments, particularly on TNFα inhibitors. As previous findings suggested that in adults T-cell responses and protective function against SARS-CoV-2 may be preserved in patients on TNFα inhibitor treatment even in case of attenuated antibody responses (24), vaccination is highly encouraged despite our findings in this group of patients. In addition, a third booster vaccination was reported to result in high neutralization efficiency and robust antibody response in adults and in children on TNFα inhibitor therapy, supporting an overall good efficacy of mRNA vaccines even when receiving combined immunosuppressive treatment (23, 25). It must be noted that the management of pediatric IBD patients with SARS-CoV-2 infection is challenging due to a number of factors including concurrent immunosuppressant treatment and an increased risk of thrombotic events (26). As both COVID-19 and IBD carry significant risk for thrombosis (27), according to current guidelines, thromboprophylaxis is to be considered in all pediatric cases with severe colitis (26). In this vulnerable group of patients, minimizing the chances of SARS-CoV-2 infection by vaccination is therefore of utmost importance. Current guidelines recommend that patients with IBD follow the same routine immunization schedule as healthy children but should avoid live vaccines if receiving immunosuppressive therapy (28). In the future, similar studies conducted on other suggested vaccination types (e.g. against influenza, human papillomavirus, etc.) might be important to help patients and caretakers to remain up-to-date and to avoid morbidity and mortality of vaccine-preventable infections.

## 5 Conclusions

In children with IBD, the extent of TG did not show a significant difference compared to healthy controls, presumably due to the fact that in most children the disease was in a quiescent stage. ETP showed a significant correlation with inflammatory parameters in IBD patients, and with PCDAI in children with CD. TG parameters, inflammatory markers and IBD disease activity scores did not increase significantly following mRNA vaccination. Previous observations of lower humoral response to anti-SARS-CoV-2 vaccination in immunosuppressed children, particularly in those on TNFα inhibitors were confirmed by our study. Our results support the safety of SARS-CoV-2 mRNA vaccination in children with IBD and may further increase confidence and reduce vaccine hesitancy in caretakers of pediatric IBD patients.

## 6 Limitations

This study must be interpreted in the context of its limitations and strengths. The sample size is limited, but it is comparable to the few studies previously published on TG in paediatric IBD patients, and it is the only study so far measuring this parameter in paediatric IBD patients pre-and post-vaccination. We did not investigate cellular response to the BNT162b2 mRNA vaccination as this was out of the scope of the study. Although IBD exacerbation rate was low at 2-6 weeks after the second dose of anti-SARS-CoV-2 vaccination in this cohort, we cannot exclude the possibility of IBD flare at a longer term. Systemic and local AEs were self-reported, which might contribute to bias. Despite these limitations, our study provides highly anticipated data regarding the safety and efficiency of the SARS-CoV-2 vaccination in pediatric IBD population and in healthy control children.

## Data availability statement

The raw data supporting the conclusions of this article will be made available by the authors, without undue reservation.

## Ethics statement

The studies involving humans were approved by Institutional Ethics Committee of the University of Debrecen and the Ethics Committee of the National Medical Research Council. The studies were conducted in accordance with the local legislation and institutional requirements. Written informed consent for participation in this study was provided by the participants’ legal guardians/next of kin.

## Author contributions

ZB: Conceptualization, Data curation, Formal Analysis, Funding acquisition, Methodology, Supervision, Writing – original draft. VS: Data curation, Investigation, Methodology, Writing – original draft. LL: Data curation, Formal Analysis, Investigation, Methodology, Writing – original draft. OK: Data curation, Investigation, Methodology, Writing – review & editing. ÉN: Investigation, Methodology, Writing – review & editing. BN: Data curation, Methodology, Writing – review & editing. RH-T: Data curation, Investigation, Methodology, Visualization, Writing – review & editing. AS: Data curation, Methodology, Formal Analysis, Writing – review & editing. MF: Data curation, Methodology, Writing – review & editing. JK: Data curation, Formal Analysis, Investigation, Writing – review & editing. TS: Data curation, Investigation, Resources, Writing – review & editing.
