# Supplementary material for: Effect of anti-SARS-CoV-2 BNT162b2 mRNA vaccination on thrombin generation in children with inflammatory bowel disease
Source: Front Immunol. 2023 Oct 30;14:1257072. doi: 10.3389/fimmu.2023.1257072 (PMC10642915; doi:10.3389/fimmu.2023.1257072)
Supplement: Supplementary file 1 [file Table_1.docx]

**Supplementary Table 1. Correlations between baseline anthropometric data, laboratory parameters and thrombin generation parameters in controls at baseline (before Pfizer-BioNTech BNT162b2 vaccine)**

|  | | **Age (y)** | **BMI (kg/m^2^)** | **hsCRP (mg/L)** | **Ferritin (µg/L)** | **WBC (G/L)** | **RBC (T/L)** | **HGB (g/L)** | **PLT (G/L)** | **PT (s)** | **APTT (s)** | **Fibrinogen  (g/L)** | |
| --- | --- | --- | --- | --- | --- | --- | --- | --- | --- | --- | --- | --- | --- |
| Thrombin generation parameters | Lag time (min) | r= -0.1907  95% CI:  -0.4267 to 0.0695  p=0.1376 | r= 0.3806  95% CI:  0.1230 to 0.5902  p=**0.0038** | r= 0.2236  95% CI:  -0.0374 to 0.4561  p=0.0832 | r= 0.1784  95% CI:  -0.0844 to 0.4180  p=0.1690 | r= 0.0989  95% CI:  -0.1620 to 0.3470  p=0.4441 | r= 0.1852  95% CI:  -0.0751 to 0.4220  p=0.1495 | r= 0.1572  95% CI:  -0.1038 to 0.3980  p=0.2222 | r= 0.0750  95% CI:  -0.1854 to 0.3256  p=0.5621 | r= 0.2041  95% CI:  -0.0556 to 0.4379  p=0.1116 | r= -0.0016  95% CI:  -0.2584 to 0.2553  p=0.9897 | r= 0.1212  95% CI:  -0.1400 to 0.3666  p=0.3480 |  |
|  | ETP (nM*min) | r= 0.2481  95% CI:  -0.0093 to 0.4747  p=0.0519 | r= 0.1569  95% CI:  -0.1184 to 0.4098  p=0.2481 | r= 0.1134  95% CI:  -0.1499 to 0.3617  p=0.3841 | r= -0.0952  95% CI:  -0.3457 to 0.1678  p=0.4651 | r= 0.1429  95% CI:  -0.1183 to 0.3855  p=0.2680 | r= -0.2323  95% CI:  -0.4615 to 0.0261  p=0.0693 | r= -0.1452  95% CI:  -0.3876 to 0.1159  p=0.2600 | r= 0.0304  95% CI:  -0.2282 to 0.2850  p=0.8144 | r= -0.4432  95% CI:  -0.6285 to -0.2103  p=**0.0003** | r= -0.3445  95% CI:  -0.5524 to -0.0961  p=**0.0061** | r= 0.2379  95% CI:  -0.0202 to 0.4662  p=0.0627 |  |
|  | Peak thrombin (nM) | r= 0.2986  95% CI:  0.0452 to 0.5159  p=**0.0184** | r= 0.0706  95% CI:  -0.2036 to 0.3345  p=0.6051 | r= 0.1624  95% CI:  -0.1008 to 0.4043  p=0.2112 | r= -0.1968  95% CI:  -0.4336 to 0.654  p=0.1285 | r= 0.1185  95% CI:  -0.1427 to 0.3642  p=0.3591 | r= -0.1579  95% CI:  -0.3986 to 0.1031  p=0.2203 | r= -0.1182  95% CI:  -0.3640 to 0.1430  p=0.3603 | r= 0.0593  95% CI:  -0.2005 to 0.3115  p=0.6466 | r= -0.4631  95% CI:  -0.6434 to -0.2341  p=**0.0002** | r= -0.5956  95% CI:  -0.7393 to -0.3999  p<**0.0001** | r= 0.2500  95% CI:  -0.0072 to 0.4763  p=0.0500 |  |
|  | Time to peak (min) | r= -0.1337  95% CI:  -0.3775 to 0.1276  p=0.3004 | r= 0.3233  95% CI:  0.0580 to 0.5459  p=**0.0151** | r= 0.0994  95% CI:  -0.1637 to 0.3494  p=0.4457 | r= 0.2505  95% CI:  -0.0090 to 0.4784  p=0.0515 | r= 0.2161  95% CI:  -0.0431 to 0.4480  p=0.0917 | r= 0.1405  95% CI:  -0.1207 to 0.3835  p=0.2760 | r= 0.1100  95% CI:  -0.1511 to 0.3567  p=0.3948 | r= 0.1190  95% CI:  -0.1422 to 0.3647  p=0.3569 | r= 0.0413  95% CI:  -0.2178 to 0.2951  p=0.7495 | r= 0.0981  95% CI:  -0.1628 to 0.3463  p=0.4477 | r= 0.1720  95% CI:  -0.0887 to 0.4107  p=1813 |  |

Spearman or Pearson correlation. APTT, activated partial thromboplastin time; BMI, body mass index; CI, confidence interval; ETP, endogenous thrombin potential; HGB, hemoglobin; hsCRP, high sensitivity C-reactive protein measurement; PT, prothrombin time; RBC, red blood cell; PLT, platelet count; WBC, white blood cell.

**Supplementary Table 2. Laboratory parameters of healthy control children before the first dose vs. 2-6 weeks after the second dose of Pfizer-BioNTech BNT162b2 vaccine**

| **Variables** | **before first dose** | **after second dose** | ***p*** |
| --- | --- | --- | --- |
| hsCRP, mg/L | 0.49 (0.49-0.76) | 0.49 (0.49-0.95) | 0.1883 |
| Iron, µmol/L | 16.4 (12.6-21.1) | 17.6 (11.8-22.3) | 0.1636 |
| Ferritin, µg/L | 41.9 (24.7-67.6) | 43.9 (25.7-75.3) | 0.1244 |
| WBC, G/L | 6.3 (5.3-7.6) | 6.2 (5.1-7.1) | 0.1197 |
| Hemoglobin, g/L | 141 ± 12 | 141 ± 13 | 0.3799 |
| Platelet count, G/L | 253 ± 53 | 250 ± 55 | 0.5010 |
| PT, s | 9.0 (8.5-9.4) | 9.0 (8.6-9.6) | 0.2849 |
| APTT, s | 29.1 ± 2.6 | 29.1 ± 2.7 | 0.8131 |
| Fibrinogen, g/L | 2.8 (2.4-3.4) | 2.7 (2.3-3.0) | 0.0408 |
| ACE2 activity, mU/L | 14.7 (12.8-16.8) | 13.3 (10.5-15.7) | 0.0424 |
| Anti-SARS-CoV-2 seropositivity, *n* (%) | 37 (60) | 61 (100) | <0.0001 |
| Anti-SARS-CoV-2 N total Ig (IgG/M), COI | 8.6 (0.1-36.5) | 4.9 (0.1-17.5) | <0.0001 |
| Anti-SARS-CoV-2 S total Ig (IgG/IgM), BAU/mL | 73.0 (0.4-206.3) | 15057 (11344-30449) | <0.0001 |

Continuous variables are expressed as mean ± SD or median (interquartile range). ACE, angiotensin-converting enzyme; APTT, activated partial thromboplastin time; COI, cut-off index; hsCRP, high sensitivity C-reactive protein measurement; PT: prothrombin time; WBC, white blood cell.

**Supplementary Table 3. Laboratory parameters and disease activity of CD patients before the first dose vs. 2-6 weeks after the second dose of Pfizer-BioNTech BNT162b2**

| **Variables** | | **before first dose** | **after second dose** | ***p*** |
| --- | --- | --- | --- | --- |
| Active disease, n (%) | | 2 (11) | 0 (0) | 0.2176 |
| Disease activity, median (IQR) | |  |  |  |
| PCDAI | | 5 (0-15) | 1 (0-13) | 0.2969 |
| hsCRP, mg/L | | 2.72 (0.94-19.38) | 0.68 (0.49-10.46) | 0.2036 |
| Iron, µmol/L | | 10.3 (4.-15.6) | 12.7 (4.5-24.0) | 0.1353 |
| Ferritin, µg/L | | 40.6 (17.1-91.1) | 31.4 (15.5-52.4) | 0.1754 |
| WBC, G/L | | 6.8 (5.7-8.1) | 6.6 (4.5-7.9) | 0.2291 |
| Hemoglobin, g/L | | 1334 ± 25 | 131 ± 21 | 0.7212 |
| Platelet count, G/L | | 282 (246-356) | 297 (250-351) | 0.9170 |
| PT, s | | 8.8 (8.5-9.4) | 9.3 (8.4-9.5) | 0.6228 |
| APTT, s | | 29.9 ± 2.9 | 30.0 ±2.5 | 0.7690 |
| Fibrinogen, g/L | | 3.5 (2.8-5.4) | 2.8 (2.3-3.9) | 0.0522 |
| ACE2 activity, mU/L | | 16.4 ± 2.8 | 15.5 ± 3.4 | 0.5276 |
| Anti-SARS-CoV-2 seropositivity, *n* (%) | 11 (64) | | 17 (100) | 0.0184 |
| Anti-SARS-CoV-2 N total Ig (IgG/M), COI | 0.2 (0.1-10.8) | | 1.5 (0.1-15.7) | 0.0205 |
| Anti-SARS-CoV-2 S total Ig (IgG/IgM), BAU/mL | 3.4 (0.4-35.4) | | 7574 (358-27495) | 0.0005 |

Continuous variables are expressed as mean ± SD or median (interquartile range). Categorical variables are indicated as number (percentage). ACE, angiotensin-converting enzyme; APTT, activated partial thromboplastin time; COI, cut-off index; hsCRP, high sensitivity C-reactive protein measurement; PCDAI, Pediatric Crohn's Disease Activity Index; PT: prothrombin time; WBC, white blood cell. Anti-SARS-CoV-2 S antibody levels were not available in 1 CD patient.

**Supplementary Table 4. Laboratory parameters and disease activity of UC patients before the first dose vs. 2-6 weeks after the second dose of Pfizer-BioNTech BNT162b2**

| **Variables** | | **before first dose** | **after second dose** | ***p*** |
| --- | --- | --- | --- | --- |
| Active disease, n (%) | | 4 (20) | 4 (20) | >0.9999 |
| Disease activity, median (IQR) | |  |  |  |
| PUCAI | | 0 (0-8) | 0 (0-10) | 0.3477 |
| hsCRP, mg/L | | 0.93 (0.49-2.88) | 0.90 (0.55-3.88) | 0.6112 |
| Iron, µmol/L | | 10.3 (5.6-16.4) | 10.6 (5.7-16.4) | 0.3344 |
| Ferritin, µg/L | | 34.2 ± 20.7 | 29.2 ± 19.1 | 0.4143 |
| WBC, G/L | | 5.7 (4.9-7.9) | 6.4 (4.5-9.1) | 0.6167 |
| Hemoglobin, g/L | | 133 (130-143) | 136 (127-145) | 0.9899 |
| Platelet count, G/L | | 301 (249-363) | 303 (254-357) | 0.4593 |
| PT, s | | 8.8 ± 0.5 | 8.7 ± 0.5 | 0.8352 |
| APTT, s | | 29.2 ± 1.7 | 29.0 ± 2.2 | 0.8161 |
| Fibrinogen, g/L | | 3.4 (3.1-3.9) | 3.3 (2.8-4.1) | 0.9799 |
| ACE2 activity, mU/L | | 17.5 (13.4-19.4) | 15.0 (12.7-17.2) | 0.1143 |
| Anti-SARS-CoV-2 IgG/IgM positivity, *n* (%) | 5 (30) | | 17 (100) | <0.0001 |
| Anti-SARS-CoV-2 N IgG/IgM, U/mL | 0.1 (0.1-11.3) | | 0.1 (0.1-8.2) | 0.0317 |
| Anti-SARS-CoV-2 S IgG/IgM, BAU/mL | 0.3 (0.3-43.7) | | 7606 (2480-12781) | 0.0002 |

Continuous variables are expressed as mean ± SD or median (interquartile range). Categorical variables are indicated as number (percentage). ACE, angiotensin-converting enzyme; APTT, activated partial thromboplastin time; COI, cut-off index; hsCRP, high sensitivity C-reactive protein measurement; PUCAI, Pediatric Ulcerative Colitis Activity Index; PT: prothrombin time; WBC, white blood cell. Anti-SARS-CoV-2 S antibody levels were not available in 3 UC patients.
